# Media coverage of Belgium’s first criminal case concerning euthanasia for psychiatric patients: A content analysis of Flemish newspapers and magazines

**DOI:** 10.3389/fpsyt.2022.1050086

**Published:** 2023-01-04

**Authors:** Marc De Hert, Erik Thys, Kirsten Catthoor, Kris Van den Broeck, Frieda Matthys, Kristof Vansteelandt, Johan Detraux

**Affiliations:** ^1^University Psychiatric Center KU Leuven, Leuven, Belgium; ^2^Department of Biomedical Sciences, Research Group Psychiatry, Center for Clinical Psychiatry, KU Leuven, Leuven, Belgium; ^3^Antwerp Health Law and Ethics Chair – AHLEC University Antwerpen, Antwerp, Belgium; ^4^Psycho-Sociaal Centrum, St.-Alexius-Elsene Vzw, Ixelles, Belgium; ^5^Ziekenhuis Netwerk Antwerpen (ZNA), Antwerp, Belgium; ^6^The Collaborative Antwerp Psychiatric Research Institute (CAPRI) and Family Medicine and Population Health (FAMPOP), University of Antwerp, Antwerp, Belgium; ^7^Flemish Psychiatric Association, Kortenberg, Belgium; ^8^Department of Psychiatry, University Hospital Brussels, Brussels, Belgium; ^9^Department of Biomedical Sciences, Research Group Psychiatry, KU Leuven, Leuven, Belgium; ^10^Department of Biomedical Sciences, Research Group Psychiatry, Public Health Psychiatry, KU Leuven, Leuven, Belgium

**Keywords:** euthanasia, end-of life, media, psychiatric disorders, legal

## Abstract

**Background:**

Belgium is one of the few countries worldwide where euthanasia on the grounds of unbearable suffering caused by a psychiatric disorder is legally possible. In April 2010 euthanasia was carried out on a 38-year-old Belgian woman with borderline personality disorder and/or autism. After a complaint by the family, three physicians were referred to the Court of Assizes on the charge of “murder by poisoning”.

**Methods:**

A content analysis of print and online news coverage of the euthanasia case in a selected sample of Flemish newspapers and magazines, published between December 1, 2019 and March 1, 2020, was conducted to analyze the prominence and framing of the euthanasia case, as well as the portrayal of key figures in this case. A quantitative analysis, as well as an in-depth qualitative analysis (with the aid of NVivo 1.0 software) was performed.

**Results:**

One thousand two hundred fifteen news articles were identified through database searching. Of these, 789 articles were included after screening for relevance and eligibility. Mean prominence scores were moderate and did not statistically significantly differ between newspapers with a different historical ideological background or form (elite versus popular). The most frequent headline topics featured legal aspects (relating to the Belgian Euthanasia Law or the course of the trial). Headlines and content of most articles (90 and 89%, respectively) did not contain an essential standpoint on the euthanasia case itself or, if they did, were neutral. Historical ideological background, nor form of newspaper (elite versus popular) significantly influenced headline tone or article direction toward the euthanasia case. Despite this, our qualitative analysis showed some subtle differences in selection, statement or tonality of reports between certain newspapers with a different historical ideological background.

**Conclusion:**

Although major Flemish newspapers and magazines generally were neutral in their coverage of the judicial case, major points of contention discussed were: the need for an evaluation and possible amendments to the existing Euthanasia Law, including a revision of the Belgian Control Commission and the system of penalties for physicians, and the absence of any consensus or guidance on how to define psychological suffering.

## Introduction

Euthanasia, defined as the intentional ending of a patient’s life through the administration of life-ending drugs by a physician at the explicit patient’s request (1–3), is only permitted by law in a small number of countries or regions worldwide. Moreover, the legal framework in these places mainly applies to terminally physically ill patients (4). Medical Aid In Dying (MAID) (defined as voluntary euthanasia and/or physician-assisted suicide) for people who are not terminally ill, such as those suffering from psychiatric disorders, is only permitted by law in Belgium, Luxembourg, the Netherlands, Switzerland, and Spain (1, 5–10).^1^ Patients receiving MAID on the grounds of unbearable suffering caused by a psychiatric disorder mostly are women and often have multiple diagnoses, in most cases including a depressive disorder or a personality disorder (1, 11, 12).

Although the number of patients with a psychiatric diagnosis requesting/receiving euthanasia has risen since 2008, it remains a limited practice in Belgium. In recent years, the percentage of euthanasia in patients with a psychiatric disorder has remained stable between 1 and 2% of the total number of euthanasia cases (5). According to the latest biannual report of the Belgian Federal Control and Evaluation Committee on Euthanasia (FCECE) (13), during the 2018–2019 period, 57 people died by euthanasia on the grounds of unbearable suffering caused by a psychiatric disorder other than dementia. Of these, 17 had a mood disorder (including major depression and bipolar disorder), 26 a personality disorder, 4 people suffered from a post-traumatic stress disorder, 7 had schizophrenia, schizotypal disorder, or a delusional disorder, and 3 were diagnosed with an autism spectrum disorder (13). During the period 2020–2021, euthanasia was performed 45 times for unbearable suffering caused by a psychiatric disorder (5).^2^

The Belgian Euthanasia Law, that came into force on the 23rd of September 2002 (14), stipulates substantive and procedural requirements that must be met for euthanasia on the grounds of unbearable suffering caused by a psychiatric disorder to be legally performed (15, 16). According to this law, patients must be legally competent and conscious at the moment of the request. The request has to be voluntary, well-considered and repeated, and not the result of any external pressure. Furthermore, the disease has to be severe and incurable, without prospect of improvement, and the psychological and/or physical suffering unbearable. The attending physician must consult two other physicians of whom one has to be a psychiatrist (15–17). Procedural requirements include that physicians have to be independent and that afterward the euthanasia has to be reported to the FCECE (15, 16, 18).

On the 27th of April 2010, euthanasia was carried out on a 38-year-old woman in the presence of her family.^3^ The procedure was approved by three physicians (two general practitioners and one psychiatrist), as the Belgian Euthanasia Law demands (15, 16). After the euthanasia had been carried out, the case was reviewed by the FCECE, which decided that the due care criteria had been met and unanimously approved the case. However, the patient’s family filed a legal complaint and claimed that not all requirements of the Euthanasia Law had been fulfilled. They argued that the patient did not suffer from a “severe and incurable disorder” as required under Belgian Euthanasia Law, as no treatment had been started for one of the diagnoses for which she had been granted euthanasia (see 5 for more details). The case was referred to the Court of Assizes and started on the 17th of January 2020. A couple of weeks later, however, the Court acquitted all three physicians for murder by poisoning. The patient’s general practitioner was acquitted because he was considered not to have been fully aware he was implicated in a euthanasia procedure, and the consulted psychiatrist because no elements were found that could justify a criminal prosecution. Although the attending physician was acquitted for murder, the Belgian Court of Cassation, after family appeal, ruled that the decision to acquit the attending physician provided by the Court of Assizes was insufficiently substantiated (19).

This case became the country’s first criminal case concerning euthanasia since it legalized the practice in 2002, and has received a lot of attention from the media, the general public, healthcare professionals, and national politicians (19). Content analysis of newspapers articles on such an important case may be a valuable method to help assess media and community opinions and advocacy (or lack of it) related to end-of-life decision making for psychiatric patients, and to see whether these differ between newspapers with different historical ideological backgrounds and forms (elite versus popular press). Belgium, a country with a population of 11.569.034 (January 1, 2022) (20), has a complex political organization. It is divided into three highly autonomous regions: Flanders (the Dutch-speaking region in the north), Wallonia (the French-speaking region in the south), and Brussels (the capital, which is officially bilingual). Finally, there is also a minority German-speaking community (in the eastern of Belgium). This aspect is relevant for our research questions, as previous research [e.g., (13, 18)] revealed significant differences in the practice, knowledge, and attitudes regarding euthanasia and its legal requirements between Flanders and Wallonia. We therefore decided to solely focus on Flemish print and online newspapers and magazines. Our research questions were fourfold:

*Research question 1:* What prominence to the euthanasia case (and end-of-life decision-making for psychiatric patients in general) was given by journalists and editors of different Flemish newspapers and magazines? Was there a difference according to the form of newspaper (elite versus popular press) or the different historical ideological backgrounds of Flemish newspapers and magazines?

*Research question 2*: What were the various angles from which Flemish newspapers and magazines approached the euthanasia case (and end-of-life decision-making for psychiatric patients in general) and did different media genres (elite versus popular press) or newspapers focus on different angles?

*Research question 3*: How did different Flemish newspapers and magazines portray the euthanasia case, the patient, her family, and the accused, and were there differences in these respects between the different media genres or newspapers?

*Research question 4*: How did Flemish newspapers and magazines frame legal, religious-political, and public and social issues related to the euthanasia case (and end-of-life decision-making for psychiatric patients in general), and were there differences in these respects among the different media genres or newspapers?

Although one of the aims of this research was to look at media coverage bias (for example to see whether certain newspapers with a particular historical ideological background choose to report more often negative or positive aspects about one party), we had no intention to examine to what extent newspaper reports contradicted with the “facts” and documents.

## Materials and methods

### Search strategy and sample selection

We used the *Gopress Academic* database^4^ to identify articles about the criminal case in eleven major Flemish newspapers, two Flemish magazines, the news service of the Flemish public broadcast (VRT) and the Belga News Agency, the leading supplier of news to all Belgian media, printed or published online between December 1, 2019 and March 1, 2020 [see also Table 1 listing the names of the selected Flemish newspapers and magazines and their daily circulation (a couple of months before the start of the court trial), historical ideological background, media group, and classification (elite–popular–free press) (21)^5^ ].

**TABLE 1:** Newspapers and magazines’ circulation, historical ideological background, and classification.

Newspaper/Magazine		Media group	Daily circulation (publ. October 2020)	Historical ideological background	Classification	Number of articles (*N* = 789)
De Morgen	DM	DPG Media	285.134	S	E	90
De Standaard	DS	Mediahuis	480.762	C	E	143
De Streekkrant	Ds	Roularta Media Group	1.124.670	X	F	0
De Tijd	DT	Roularta Media Group	216.837	X	E	16
De Zondag	DZ	Roularta Media Group	1.434.292	X	F	33
Gazet van Antwerpen	GvA	Mediahuis	455.150	C	Po	53
Het Belang van Limburg	BvL	Mediahuis	423.246	C	Po	17
Het Laatste Nieuws + De Nieuwe Gazet	HLN	DPG Media	1.481.577	L	Po	123
Het Nieuwsblad + De Gentenaar	NB	Mediahuis	1.089.146	C	Po	79
Krant van West-Vlaanderen	KW	Roularta Media Group	389.624	X	Po	5
Metro NL	MNL		372.152	X	F	29
Knack Magazine	KM	Roularta Media Group	451.199	P	E	52
Humo	H	DPG Media	527.043	X	Po	7
Flemish public broadcast/the Belga news agency		Mediahuis and Groupe Rossel.				
VRT online	VRT	VRT	N/A	X	E	32
Belga	BE	Belga	N/A	X	E	110

C, Christian; E, elite; F, free; P, plural; Po, popular; L, liberal; S, socialist; N/A, not applicable; X, none.

A combination of the keyword “euthanasia” with the patient’s given name and/or surname of the patient was used to select the articles. We included regular news reports, letter(s) to the editor, columns, expert opinions, comments and other stories/articles providing background information. Selected articles had to contain at least 150 words, had to be published in newspapers or magazines that were selected for inclusion, and had to be written in Dutch. Thus, articles where the euthanasia case was only mentioned briefly or articles written in French were excluded. The electronic search was complemented by manual searches in paper archives.

### Coding procedure

All selected articles were systematically coded by JD based on the instructions contained in a coding sheet (see Supplementary material 1). To ensure reliable coding, we extensively pretested the coding sheet during three stages, whereby five authors each time reviewed a sample of articles and met online to discuss the coding system. During the first stage a sample of twenty randomly chosen articles were independently coded by the authors JD, MDH, KC, KVDB, and FM to determine whether the constructed categories “topics featured in the headline,” “headline tone,” and “article direction toward the euthanasia case” were adequate. Coders were explicitly instructed to assess the tone of an article’s headline before they read the article’s text; so, the full text’s coding could not influence the judgment of the headline. On the basis of this coding, the sheet was adapted and a new sample of ten randomly chosen articles were coded by the same five authors. Finally, a sample of five randomly chosen articles were coded by these authors, after which the coding sheet was finalized. JD used the final coding sheet to code all selected articles.

All articles were coded according to the following categories:

(1) Descriptive variables
(a) **Name** of newspaper, magazine, or online news services of the Flemish public broadcast (VRT) or the Belga News Agency.
(b) **Type** of article: regular news reports, letter(s) to the editor, columns, expert opinions, and other stories/articles providing background information or general commentary on the issue.
(c) **Author and title** of article and **gender** (M/F) of the author.
(d) **Publication date.**

(2) Prominence score

The “prominence score” reflects the degree of importance of each article given by editors (23). In our study, article prominence was assessed, using the prominence scoring classification system of Pollock and Yulis (24) (see Table 2).

**TABLE 2:** Prominence score for coding databases (24).

Dimension	4	3	2	1
Placement	Front page of first section	Front page of inside section	Inside first section	Other
Headline size (in number of words)	≤10	8–9	6–7	≥5
Article length (in number of words)	≤1,000	750–999	500–749	150–499
Photos/Graphics	Two photos or graphics	One photo or graphic		

A score ranging from 3 to 16 was assigned based on four factors: placement in the newspaper (front page, first section, etc.), headline word count, article length (number of words), and any accompanying photographs and/or graphics (with/without caption). Articles receiving a higher number of points are thought to obtain a higher attention score and are [thought to be] more likely to be read (24).

(3) Topic(s) featured in the headline

Topic(s) featured in the headline were classified according to one of the following categories: legal aspects, ethical aspects, personal or familial aspects, political aspects, controversial aspects, medical or scientific aspects, public and social aspects, religious aspects, historical aspects, other (see Table 3). If the headline featured several aspects it was coded as many times as appropriate to the data (rich coding strategy).

**TABLE 3:** Headline topics.

Headline topics	Examples
Legal aspects	“Euthanasia trial could break the law”
Ethical aspects	“A public trial of this emotional case feels like completely inappropriate voyeurism”
Personal or familial aspects	“Drugs, prostitution, a lot of beatings. And a persistent death wish”
Political aspects	“Painful process puts politicians to work”
Controversial aspects	“Physicians are there to heal us, not to help us die”
Medical or scientific aspects	“Autism? Borderline? Both?”
Public and social aspects	“GPs withdraw from files relating to euthanasia”
Religious aspects	“Nobody expects the Spanish Inquisition”
Historical aspects	“These were the early years of euthanasia for psychological suffering”
Other	“Our opinion”

(4) Headline tone

We assessed the general tone of the headline (as positive, neutral, or negative). If it also contained an essential standpoint on the euthanasia case itself, it was also assessed to determine neutrality or bias for or against the euthanasia case (i.e., containing positive or negative wording toward the euthanasia death of the patient or the people that euthanized the patient). Take for example the headline “Jef Vermassen *(attorney of the consulted psychiatrist)* cynical about the judicial process: ‘This woman should be tried for serial murder”’. Generally speaking, this headline is negative (as it is written with negative wording), but with regard to the euthanasia case it is positive (because of the cynical nature of the comment, it is clearly intended to be supportive for the psychiatrist who wrote the advice for euthanasia).

(5) Article direction toward the euthanasia case (favorable, balanced/neutral, unfavorable, or not applicable).

We also assessed the article direction toward the euthanasia case. Articles simply representing or reflecting the facts of the case, displaying both sides of the debate over the euthanasia case in approximately equal measure, and/or stating that this issue is not for any of us to judge, were considered neutral. Articles more than average making critical remarks about the patient’s family, their attorneys, opponents of euthanasia for psychiatric patients and/or being sympathetic to the organizations that support euthanasia for psychiatric patients were classified as favorable. Articles making critical remarks about the physicians (or their attorneys) involved in the euthanasia case, advocates of euthanasia for psychiatric patients and/or being aloof from organizations that support euthanasia for psychiatric patients were deemed to be unfavorable.

### Analyses

Frequencies and percentages were calculated for each aspect of interest. For “headline tone” (in general and specifically toward the euthanasia case) and “article direction” chi-squared tests or Fisher’s exact tests, when appropriate, were performed to test differences between Flemish newspapers and magazines according to their historical ideological background and newspaper form. For “topic(s) featured in the headline” no chi-squared testing was performed as we did not obtain a set of mutually exclusive categories due to our decision to follow a “rich coding strategy.”

Three separate analysis of variance (ANOVA) analyses were performed to see whether there were differences in “mean prominence scores” in terms of respectively (a) name of newspaper, (b) historical ideological background (Christian, socialist, liberal, and plural), and (c) form of newspaper (elite vs. popular vs. free). In these analyses, when the overall test was significant, the different categories were compared using *post hoc* Tukey pairwise comparison tests. As there is currently no consensus within the literature on how to monitor online article prominence (25) (most online versions of newspaper articles are updated constantly as new content becomes available, are harder to register, more fluid, and increasingly do not contain the same content as the print editions), only print articles were coded by using this classification system.

A qualitative analysis, using Nvivo 1.0 (Mac version), was conducted to examine the content of print newspaper articles. This method was chosen to thoroughly analyze the media portrayal of all persons involved in the euthanasia of the patient and to identify the major issues or debates emerging during court proceedings. One of the authors (JD) developed the coding framework (see Supplementary material 1). This framework was then refined through discussions by the larger research team, and modified slightly thereafter before it was applied by JD to the data set of newspaper articles. For the same reasons as mentioned above concerning the “prominence score” analyses, only print newspaper articles were used for this qualitative analysis.

We used mixed deductive and inductive coding to analyze the data. For this analysis, the coding structure applied to all print articles included: (1) the media portrayal of major persons involved in the euthanasia case [i.e., the patient, the consulted psychiatrist, the attending physician, the general practitioner, and the family of the patient (father, mother, two sisters, and one brother)], trying to answer the following question: “How are these persons portrayed in the selected newspaper and magazine articles and are there differences in these respects among the different media genres, historical ideological backgrounds, and newspapers?”; (2) legal aspects: discussion focusing on gaps between law and practice; (3) public-social consequences; and (4) religious-political polemics: emphasizing the conflict between conservatives and progressives.

## Results

### Descriptive characteristics

**FIGURE 1:**
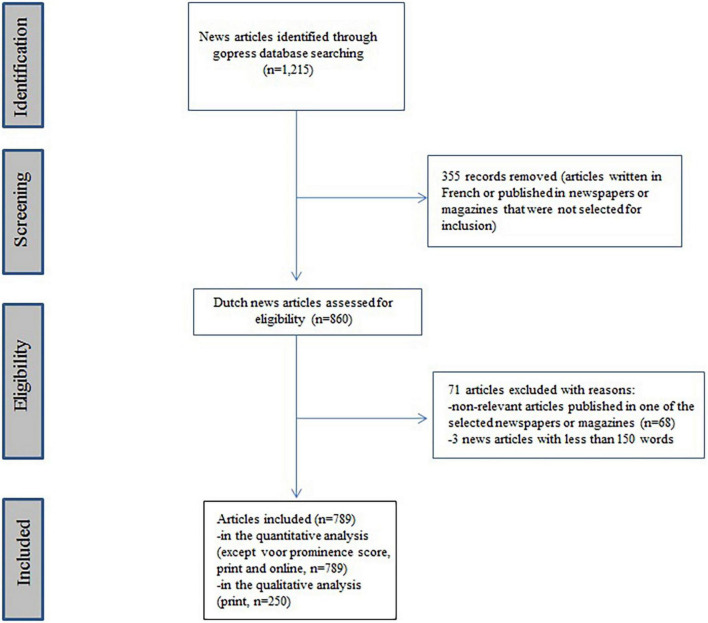
Flowchart of literature search.

Our search yielded 1,215 articles (see Supplementary material 2). Articles were screened and those that were written in French, were published in newspapers or magazines that were not selected for inclusion (*n* = 355), were non-relevant, or did not minimally discuss the euthanasia case (*n* = 71) were discarded from the sample (for the flowchart, see Figure 1).

**FIGURE 2:**
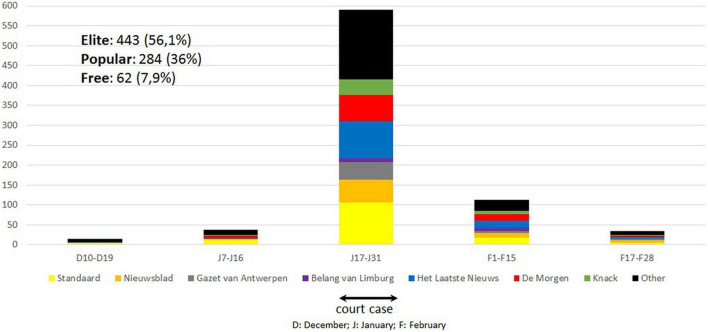
Number of news articles published during the time period December 2019-March 2020.

We retrieved a total of 789 relevant articles, published online or in print between the 1st of December 2019 and the 1st of March 2020. Figure 2 gives an overview of the number of news articles published during the time period December 2019–March 2020. The majority of the coverage (73.3%) occurred between January 20 and February 1, 2020 (with a peak of 125 articles on January 31). Most articles were regular news reports (71.1%).

***Research question 1:*** **What prominence to the euthanasia case (and end-of-life decision-making for psychiatric patients in general) was given by journalists and editors of different Flemish newspapers and magazines? Was there a difference according to the form of newspaper (popular vs. elite press) or the different historical ideological backgrounds of Flemish newspapers and magazines?**

**FIGURE 3:**
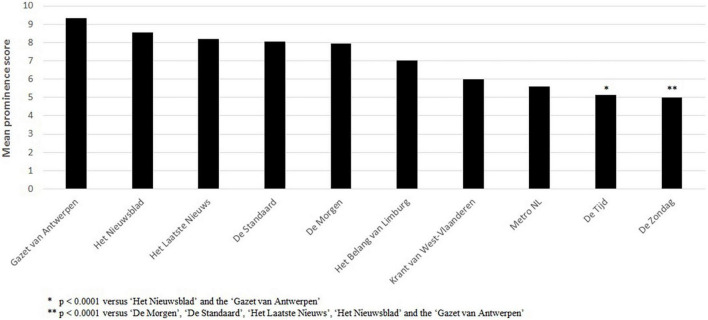
Mean prominence score per newspaper.

Prominence mean scores were moderate (roughly between 7 and 9 for the most important non-free Flemish newspapers). In terms of mean prominence scores for the selected newspapers, results indicated that there were statistically significant differences between some newspapers [*F*(10,210) = 191.07, *p* < 0.0001; Figure 3]. ‘De Zondag’ (X,F), the largest free Sunday newspaper in the Flemish-speaking part of Belgium and having the lowest mean prominence score (5.0), was statistically significantly different from ‘De Morgen’ (S,E) (7.94), ‘De Standaard’ (C,E) (8.06), ‘Het Laatste Nieuws’ (L,Po) (8.20), ‘Het Nieuwsblad’ (C,Po) (8.54), and the ‘Gazet van Antwerpen’ (C,Po) (9.32) (all *p* ≤ 0.01). In addition, ‘De Tijd’ (X,E), a Belgian newspaper that mainly focuses on business and economics, had a statistically significantly lower mean prominence score (5.13), compared to ‘Het Nieuwsblad’ (C,Po), and the ‘Gazet van Antwerpen’ (C,Po) (all *p* ≤ 0.05). All other newspapers did not statistically significantly differ from one another in terms of mean prominence score. There were statistically significant differences in terms of ideology (mean prominence score for newspapers with no clear ideology = 5.53 vs. newspapers with an historical ideological background = 8.20, *F*(5,210) = 357.33, *p* < 0.0001), and in terms of form of newspaper between ‘free’ and other newspaper forms [*F*(3,217) = 602.21, *p* < 0.0001, ‘free’, ‘elite’, and ‘popular’ newspapers had mean prominence scores of respectively 5.14 (se = 0.60), 7.75 (se = 0.29), and 8.32 (se = 0.26)]. There were, however, no statistically significant differences in mean prominence scores between newspapers with a historical ‘Christian’, ‘liberal’, ‘socialist’, or ‘plural’ ideology, or between ‘elite’ and ‘popular’ newspapers.

***Research question 2***: **What were the various angles from which Flemish newspapers and magazines approached the euthanasia case (and end-of-life decision-making for psychiatric patients in general) and did different media genres (popular vs. elite press) or newspapers focus on different angles?**

**FIGURE 4:**
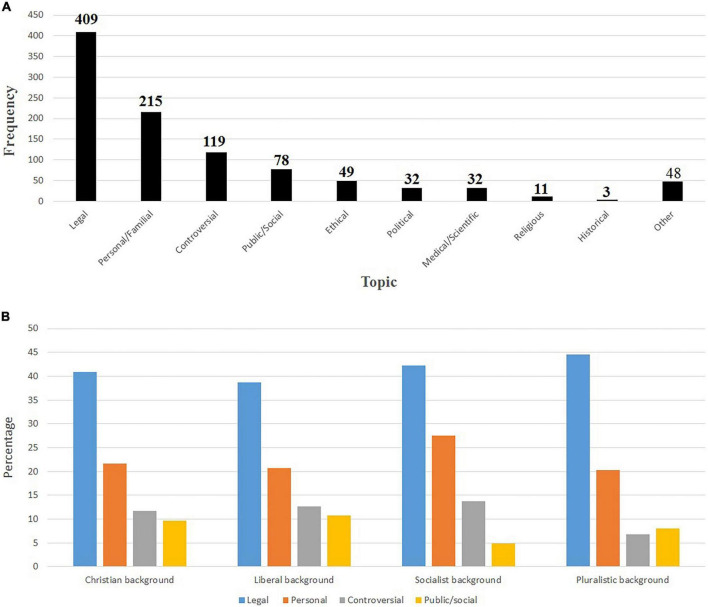
**(A)** Topic(s) featured in the headline. **(B)** Most important angles to approach the euthanasia case according to historical ideological background of newspapers and magazines.

Figure 4A presents the most common topics found in headlines of newspaper articles covering the euthanasia case. Headline topics most frequently focused on the legal aspects of the case (*n* = 409, of which 138 referred to the Euthanasia Law itself, and 271 to the course of the trial), aspects relating to the private or family sphere or personal feelings (*n* = 215), and controversial aspects (persons or topics that are the subject of intense public argument, disagreement, or disapproval) (*n* = 119). Figure 4B presents the most important angles to approach the euthanasia case according to historical ideological background of newspapers and magazines.

***Research question 3:*** **How did different Flemish newspapers and magazines portray the euthanasia case, the patient, her family, and the accused, and are there differences in these respects between the different media genres or newspapers?**

### Quantitative analysis

Generally speaking, a minority of headlines (11%) had a positive tone (vs. 47% neutral, and 42% negative). Most of the headlines (90%) did not contain an essential standpoint on the euthanasia case itself or, if they did, were neutral. Likewise, the content of most articles (89%) did not contain an essential standpoint on, or were neutral about the euthanasia case itself (see Supplementary material 3). None of the included newspapers or magazines were statistically significantly more likely to use positive or negative wording in their headlines (χ^2^ = 17.9497, *df* = 10, *p* = 0.0558) or to report positive or negative aspects toward the euthanasia case or euthanasia for psychiatric patients (χ^2^ = 4.0782, *df* = 3, *p* = 0.2531) in these headlines. There were no statistically significant differences in terms of ideology (χ^2^ = 7.7818, *df* = 4, *p* = 0.0999 for general headline tone; χ^2^ = 2.7394, *df* = 3, *p* = 0.4336 for headline tone toward euthanasia case or euthanasia for psychiatric patients) or newspaper form (χ^2^ = 7.2106, *df* = 4, *p* = 0.1252 for general headline tone; χ^2^ = 5.0148, *df* = 6, *p* = 0.5419 for headline tone toward euthanasia case or euthanasia for psychiatric patients).

No statistically significant differences between newspapers and magazines were detected in reporting favorable or unfavorable aspects toward the euthanasia case or euthanasia for psychiatric patients in their articles (χ^2^ = 2.9333, *df* = 3, *p* = 0.4020). There were no statistically significant differences in terms of ideology (χ^2^ = 0.7903, *df* = 3, *p* = 0.8518). However, a statistically significant difference was found in terms of newspaper form: ‘free’ press articles never contained favorable or unfavorable aspects toward the euthanasia case or euthanasia for psychiatric patients, compared to ‘popular’ or ‘elite’ press (‘free’ vs. ‘popular’ and ‘elite’, χ^2^ = 23.2053, *df* = 6, *p* = 0.0007).

### Qualitative analysis: Media portrayal of the persons involved in the euthanasia case

#### The patient

The emphasis of most newspaper articles clearly lied on a detailed, dramatic, and emotional description of the patient’s life, with a particular focus on childhood trauma, making her **a tragic figure** both in terms of her illness and of her family history. According to these articles, the patient had been expressing a death wish from the age of 14. She had severe emotional and behavioral problems (depressions, anxiety), leading to multiple psychiatric hospitalizations. She often attempted suicide and spent 10 days in a coma after a major suicide attempt in 1997, causing a long-lasting physical disability.

*“Prostitution, a lot of beatings and a persistent death wish: euthanasia process exposes (name of the patient)’s hard life course.” (Het Nieuwsblad, January 18, 2020).*

*“Indeed. So much misery, such a death wish – I don’t think I could’ve handled it.” (De Standaard, January 25, 2020).*

Nevertheless, some newspapers balanced this picture by providing articles describing her as an **ordinary or even magnificent person** enjoying life, being generous, helping other people, or as a driven person with dreams like everybody else. This seemed particularly the case for *De Morgen* (S,E) by giving almost equal attention to both aspects. *Het Laatste Nieuws* (L,Po) and *De Standaard* (C,E), however, mainly focused on the downfall of the patient. *Het Belang van Limburg* (C,Po) only talked about her tragic life course without saying anything about who she was as a person. De *Gazet van Antwerpen* (C,E) and *Het Nieuwsblad* (C,Po) occupied an intermediate position.

*“(Name of the patient) was a helpful, intelligent woman who even gave her own bed to people who needed help”, friends told about (name of the patient). It also happened that the bell rang and that one of her acquaintances was standing at the door, blind drunk. She put her in her bed and slept on the couch herself. But she thought that was normal. ‘That person can no longer drive her car, can she?’ (Name of the patient) was generous. She had a heart of gold. She hated injustice and saw it as her duty to help those in need” (Gazet van Antwerpen, January 27, 2020).*

Few newspaper articles described the patient as a **heroic, courageous warrior,** leaving a legacy of choice.

*“Make (name of the patient) the Jeanne d’Arc of euthanasia” (De Morgen, January 30, 2020*).

Equally, little attention had been given to the perspective of the civil parties, making the patient **a victim** of psychiatry and advocate of euthanasia, stating that “she did not want to die”, that “this is not what she really wanted”, that “there was a pressure to ask for euthanasia”, and that “she might have changed her mind”.

*“She was deliberately pushed toward death. (Name of the patient) has ended up in the web of the psychiatry of death” (Gazet van Antwerpen, January 18, 2020).*

#### The consulted psychiatrist

All newspapers paid attention to the consulted psychiatrist’s profile as it was presented by the attorneys of the civil parties, as someone **manipulating and luring suicidal people into their own death**, or as the **euthanasia crusader** who too easily approves euthanasia requests from patients with mental illness. She was described as “a guru”, “psychiatrist of death”, “doctor death”, or as “someone not taking her responsibility”. Once in the hands of this psychiatrist you were “on a highway to euthanasia”. She sat “behind the wheel of death, leading people to their death”.

*“(Name of the patient) fell under the spell of (name of the consulted psychiatrist). She became a guru, a new mother. And she led her to her death” (Gazet van Antwerpen, January 18, 2020).*

Civil parties also mentioned that a book would be written about the patient and her euthanasia request (the patient indeed appeared under a pseudonym in a book by the consulted psychiatrist that was published in 2011) and that a photo essay, commissioned by the consulted psychiatrist, was developed by a student during her euthanasia request, making the pressure on the patient’s shoulders to continue with the euthanasia immense.

*“The sisters are convinced that (name of the consulted psychiatrist) wanted to make (name of the patient) a fitting figurehead for the euthanasia of people suffering from mental illness” (De Morgen, January 22, 2020).*

However, with the exception of *De Standaard* (C,E), all newspapers clearly balanced this image, emphasizing her as someone who actually **tries to save people** by keeping open the option for life (patients knowing that their suffering can come to an end if they want, may feel a relief, which itself can be life-saving), or by offering alternative solutions. She was also depicted as “merciful as Father Damien”, as “someone providing housing to the most vulnerable social groups”, as **“a courageous warrior”** making her heroic, as a “respected psychiatrist”, and as a “responsible person”.

*“Witnesses paint a very different picture of the psychiatrist. Pushed to death? She just saved our daughter” (Gazet van Antwerpen, January 18, 2020).*

Finally, some newspapers [i.e., *De Gazet van Antwerpen* (C,E) and *Het Nieuwsblad* (C,Po)] described the consulted psychiatrist as someone who likes to see herself as a **pioneer of euthanasia** for people struggling with mental illness for years.

#### The attending physician

All newspapers [particularly *Het Laatste Nieuws* (L,Po)] consistently described the attending physician as a nervous, clumsy physician. The euthanasia procedure was said to be “a mess”, and the way he handled it “didn’t deserve a beauty prize” and was “not a nice ending”. One newspaper article even called him Mr. Bean. The physician himself admitted that he “made some annoying mistakes, but nothing more”, because “he was stressed”.

According to one newspaper, the attending physician had never performed a euthanasia for psychological suffering before, and had not completed his end-of-life care training course. Several newspapers mentioned that during the euthanasia procedure he asked the patient’s father to hold the needle in her arm because he had forgotten to bring plasters. It was told that he did not have a stand for the infusion, and that the bag fell onto the patient’s face just before she died. His mobile phone was ringing all the time, and he likened her death to that of a pet that is in pain and is having a shot. After her death, he asked the parents if they wanted to use the stethoscope to check whether her heart had actually stopped beating.

Finally, the attending physician was said to have sent the registration form to the FCECE 51 days after the euthanasia was performed (while the Belgian law requires to send the registration form to the commission within four working days). Interestingly, *De Morgen* (S,E) was the only newspaper that, next to these descriptions, explicitly stated several times that this does not make him a murderer, and that what he was doing was not illegal. *De Morgen* (S,E) is also the only newspaper calling him courageous, presenting him as someone who did take his responsibility and acted decisively, because at that time “he was the only one in the neighborhood who followed the end-of-life care training course”.

Some newspapers [i.e., *De Morgen* (S,E) and *Het Nieuwsblad* (C,Po)] reported extensively on the character sketch of the attending physician in the bill of indictment. His background was highlighted in these articles. In fact, he had been in court before for various offenses, including drunk driving and sex offences. However, according to these newspapers by using this character sketch “some people wanted to put him in a bad light” with the purpose to present him to the jury as a “sadistic murderer, as someone with a bad reputation”.

*“You can already sense that the trial will primarily revolve around (name of the attending physician). Judging by the character sketch in the indictment, he will…be presented to the jury as a sardonic grinning Doctor Death with a skull on his physician’s bag. Or as a physician who at least doesn’t have a great reputation” (De Morgen, January 11, 2020).*

*“The apparent wish of some parties in this process: that (name of the attending physician) lingers in the air until the jury has to deliberate on his guilt in this euthanasia case” (Het Nieuwsblad, January 25, 2020).*

#### The (consulted) general practitioner

Most newspaper articles described the general practitioner as **a good family practitioner taking care of his patients** in the community, and **as a simple man who has been set up**. The newspaper most often focusing on both these aspects was *Het Laatste Nieuws* (L,Po), followed by *De Standaard* (C,E) and *De Morgen* (S,E). The general practitioner, who was principally opposed to euthanasia and actually did not want the patient to get euthanasia, painted himself as the victim of a charming and manipulative young woman he cared about deeply and who had been his patient for over 10 years. He also said he felt betrayed by the patient’s family (for having made hidden recordings while pretending they wanted to speak to him to facilitate their grief), and by his colleagues (particularly the attending physician who fooled him). According to some news reports (e.g., *De Standaard*, January 20, 2020), on the evening of the euthanasia, the attending physician dropped by, asking the general practitioner to sign a paper (which was not intended to be an advice), saying he was on his way to “a meeting” with the patient and her family. On the paper it was written that while he supported her request, at the same time regretted her decision. The patient herself had told him it was only an administrative application and that the attending physician would come to pick it up. The general practitioner also thought that the request first had to be approved by the FCECE. He was aghast when a couple of days later he got the obituary and learned that the patient had been euthanized. Interestingly, given the above mentioned circumstances this meant that he did not actually provide the advice which was needed to make the procedure legal.

*“It was just a written note. I thought it was necessary for the application to the euthanasia committee. (Name of the attending physician) came to get it at my cabinet (.). I didn’t know the euthanasia would happen that day. I fell off my chair when I received the obituary” (Knack VIP, January 20, 2020).*

#### The patient’s family

All newspapers consistently gave attention to the description of the patient’s family as **people who did not take enough care of, or did not pay enough attention to the patient**. Sometimes they identified them as “a seriously **dysfunctional**, **wounded, traumatized family** with very little empathy and respect for others”, or as **“inquisitors”**. However, *Het Laatste Nieuws*, was the only newspaper overemphasizing this picture.

*“Sometimes an eyebrow raised, for example when the chairman asked (name of one of the patient’s sisters): “Did you know why (name of the patient)’s last relationship broke down?” Silence. Brief reply. You would expect sisters who are ‘exceptionally close’ to know more about each other, some thought. Did (name of one of the patient’s sisters) know why her sister was so unhappy? Tried suicide? Not in detail, it turned out. Same with her brother (name of the brother)” (Het Laatste Nieuws, January 22, 2020).*

Although sometimes newspaper articles cited the attorneys identifying the patient’s family as **victims of psychiatry and advocates of euthanasia**, many newspapers published letters to the editor where the family was even getting “slaughtered” by the general public who was blaming them of starting the criminal case with the wrong intention.

*“I fear that there is manipulation to roll back the law. (Name of the patient)’s family has fallen into a trap” (De Morgen, January 28, 2020).*

***Research question 4:*** **How did Flemish newspapers and magazines frame legal, religious-political, and public and social issues related to the euthanasia case (and end-of-life decision-making for psychiatric patients in general), and were there differences in these respects among the different media genres/newspapers?**

### Legal aspects

#### Incurable disease and unbearable suffering

An incurable disease (i.e., being in a medical condition, without prospect of improvement, and with the absence of possible curative interventions) and the patient’s constant and unbearable suffering that cannot be alleviated (which is up to the patient to be judged and for which no generally accepted definition or operationalization is available) are essential criteria for granting euthanasia.

The consulted psychiatrist, as well as the court-appointed expert committee claimed that all reasonable treatment options for borderline personality disorder (BPD) had been tried. However, after the patient underwent additional diagnostic testing during the evaluation of her request she was diagnosed with autism (Asperger Syndrome) only several weeks before her death. The court-appointed expert committee found it highly problematic that for this new diagnosis no treatment had been tried, although an offer made by the consulted psychiatrist had been refused by the patient.

Although the family acknowledged her severe mental problems in the past, according to them the patient was not incurably ill, but suffering from the stress of a broken relationship (the euthanasia request of the patient followed the breakup of a relationship mid-2009 which confronted her with prior problems in coping with life and a perceived impossibility to maintain relationships) (5). Moreover, she had not had residential psychiatric treatment for many years.

All the above mentioned aspects were mainly highlighted by the newspapers *De Morgen* (S,E) and *Het Laatste Nieuws* (L,Po). *Het Laatste Nieuws* (L,Po) was the only newspaper that extensively discussed the testimony of the patient’s psychologist. She was the only mental health professional who still followed the patient since her last hospital admission. She worked as a therapist in a community mental health center and provided supportive counseling to the patient until the end of 2009 (in 2010 the psychologist only occasionally had contact by phone). Her testimony would turn out to be of crucial importance for the final conclusion of the court-appointed experts, because they reconsidered their statement after this testimony. Although they *initially* found it highly problematic that no treatment had been started for the new diagnosis (although it had been proposed, but refused by the patient), after hearing the testimony of the psychotherapist (who had no specific expertise on either BPD or autism), the court-appointed experts concluded that the patient’s psychological suffering, even when starting treatment for this new diagnosis, probably would be irremediable. The Flemish newspaper *De Standaard* (C,E) clearly had problems with this turnaround. During the trial the newspaper explicitly put it in the following way:

*“After almost 20 years of legal regulation, euthanasia has become so accepted that physicians and patients deal with it more freely. It is no longer just an emergency brake for when no treatment options are left. Nowadays, patients also consider euthanasia as an early exit if they no longer consider further treatment convenient” (De Standaard, January 13, 2020).*

Interestingly, while *Het Nieuwsblad* (C,Po), *De Gazet van Antwerpen* (C,Po), *De Morgen* (S,E), and *Het Laatste Nieuws* (L,Po) all extensively discussed the testimony of one of the experts in support of the patient’s family, stating that *“it can never be asserted that a psychiatric patient’s case is hopeless,”* and that *“borderline [personality disorder] always is remediable”*, the former two newspapers did it in a neutral way, while the latter two edited it with personal comments. *De Morgen* (S,E) stated in one of its newspaper articles that the expert’s statement *“that you can heal every psychiatric disorder”* is a dogma and that if this is the case *“you simply can abolish the euthanasia law for psychological suffering”.* After discussing the testimony of this expert, the journalist of *Het Laatste Nieuws* (L,Po) added laconically that *“the defense attorneys sneered at such arguments”*.

The newspaper *De Standaard* (C,E) most extensively spoke about the medical-scientific discussions among experts of the parties on the correct diagnosis for the patient: “BPD, autism or both?”, emphasizing in this way the problematic nature of euthanasia based on a psychiatric diagnosis. Moreover, the newspaper also published some stories about how being diagnosed with autism saved a person’s life. *De Morgen* (S,E) barely mentioned the issue, while *De Gazet van Antwerpen* (C,Po), *Het Nieuwsblad* (C,Po), and *Het Laatste Nieuws* (L,Po) occupied an intermediate position.

#### Functioning and reporting to the federal control and evaluation commission for euthanasia

Compared to newspapers with a historical Christian ideology, socialist and liberal newspapers paid a lot of attention to the main attorney who represented the patient’s family, well known to have a very strong conservative ideology and controversial standpoints. During the course of the trial he was forced to step down when a conflict of interest arose. In fact, the chairman of the FCECE revealed that the attorney had been a non-voting member of the FCECE when the patient’s case was approved. The attorney himself described the issue as *“a storm in a teacup”*.

Less attention has been given by newspapers to the lack of neutrality on the part of the chairman of the FCECE when evaluating the registration form of the attending physician. The report that needs to be submitted to the FCECE consists of an anonymous part and a part with the identifying data of the persons involved. However, in the case of the patient, the anonymous part contained identifying data.

“(Name of the chairman of the FCECE) *was attacked by (name of the attorney for the parents), attorney for the parents of (name of the patient). The attorney projected the file as it arrived at the FCECE. These reports normally are anonymized, but (name of the patient)’ first name, as well as that of psychiatrist (name of the consulted psychiatrist) appeared” (De Morgen, January 23, 2020).*

*“When civil parties showed that anonymous part, it turned out that what it said was quite recognizable: the name (name of the patient) was mentioned three times, and her situation was also described in great detail” (De Standaard, January 23, 2020).*

In this regard, it is important to know that a few days before her death, the chairman of the FCECE, who equally took part in the evaluation of the registration form submitted, received a call from the patient’s father who was dissatisfied with the state of affairs, after which he contacted the consulted psychiatrist. A few months after the euthanasia was performed he was called by one of the patient’s sisters who complained about the procedure. Two days before the evaluation of the registration form, the chairman of the FCECE and the physicians had made an attempt at reconciliation with the family. Taking all these things together, the chairman therefore must have known who it was when evaluating the “anonymous” part of the form.

Remarkably, the problematic functioning of the FCECE (*a posteriori* control, the issue of underreporting of euthanasia cases, lack of transparency, not being able to perform their job thoroughly) has only been extensively discussed in the newspaper *De Standaard* (C,E).

*“The FCECE hardly contributed to the careful handling of the law. A retrospective control - the commission’s task - cannot prevent mistakes. At most you can document it afterward, and then based on what the attending physician wanted to disclose in writing” (De Standaard January 21, 2020).*

One of its newspaper articles mentioned that the FCECE is *“like a conveyor belt: the public prosecutor calculated that the FCECE spends on average 45 s per file”*.

#### Independence

Mainly three newspapers focused on the aspect of independence of the involved physicians: *De Standaard* (C,E), *Het Laatste Nieuws* (L,Po), and *De Morgen* (S,E). With the exception of the latter, the other two newspapers very critically discussed this issue. According to the Belgian Euthanasia Law, the attending physician needs to consult two other physicians, who should be independent of both the patient and the attending physician. Although “independence” is not defined in the Euthanasia Law, it is usually interpreted as the absence of a regular therapeutic relationship between the patient and the consulted physician, or as the absence of family ties or hierarchical relation between the patient and the consulted physician (26). Anonymous reporting makes this virtually impossible to check by the FCECE.

During the trial the opinions of the experts of the parties differed substantially with respect to the aspect of independence of the patient’s general practitioner. According to the court-appointed experts he was not independent because the patient visited him every 2 weeks for a consultation and because he was already 9 years her treating general practitioner. According to the chairman of the FCECE, however, a treating general practitioner can be regarded as an independent, advising physician if he says he does not want to be involved with this euthanasia (i.e., perform the euthanasia) and refers the person to another physician (something the general practitioner actually did).

Reporting differences were noted when comparing newspapers. According to *De Morgen* (S,E) members of the expert commission stated that all physicians were independent of each other and that, with the exception of the patient’s general practitioner, the physicians also were independent from the patient. *De Standaard* (C,E), generally somewhat more critical for the accused physicians, however, mentioned that the expert commission stated that the consulted psychiatrist was independent at the beginning, but probably lost her independency during treatment of the patient. In fact, during the trial it was suggested that the consulted psychiatrist also treated the patient as she occasionally prescribed a sedative or hypnotic for her. Moreover, the importance of transference and counter-transference phenomena was also stressed by various experts of the parties and court-appointed commission.

### Religious-political polemics

One of the key themes emerging from the qualitative analysis was “religious-political polemics”, despite the fact that only 4.3% of the topic(s) featured in the headlines could be classified as religious and/or political, and that the chairman of the court did succeed in avoiding *“a political-religious process about the role of the Catholic Church and freemasonry”*.

*“This will be a political-religious trial with the euthanasia law at stake. So a show trial” (Het Laatste Nieuws, December 14, 2019).*

*“Defense attorneys suspect a reactionary plot to roll back the law and intimidate physicians” (De Standaard, January 21, 2020).*

At the start, during and after the court case one of the attorneys, representing the attending physician, wondered aloud whether the superior general of the Catholic Congregation Brothers of Charity and close associate of Pope Francis (at the insistence of “Rome”, “the Catholic Lobby,” “the hidden counterparty”) put pressure on the public prosecutor’s office (*ministère public/openbaar ministerie*). In fact, although by the end of 2016 a court in Dendermonde first refused to press charges against the three physicians, this verdict was overturned in 2017 by a court in Ghent, which sent the case to the Court of Assizes. According to the attorney his client had heard from a physician friend that the superior general had made approaches to the Ghent prosecution service intended to convince them to prosecute. Moreover, the decision to prosecute the three physicians came at the moment that The Brothers of Charity had decided to allow physicians to perform euthanasia for psychiatric patients under certain conditions within their facilities. After protest of the superior general in 2017 the Vatican Congregation for the Doctrine of the Faith ruled in 2020 that the pro-euthanasia position of the Provincial Association of the Brothers of Charity was incompatible with church teaching on the inviolability of human life, and therefore stripped their Belgian facilities for psychiatric patients of their Catholic status. Finally, the attorneys representing the interests of the patient’s family were depicted as devout and prominent “Catholic intellectuals” and fighters against the extension of the Euthanasia Law, one defending in 2010 the cardinal archbishop of Brussels and Mechelen, the other defending another well-known cardinal, both in matters relating to child abuse by clergy of the Roman Catholic Church.

Although no proof has been provided to date for these accusations, several newspapers with a historical non-Christian ideological background clearly were very skeptical on these points, and followed these accusations.

*“The decision that has been made that the case is to be tried in the Assizes court is a very strange turnaround! It is therefore not surprising that many believe that the whole case was brought as a legal attack on the euthanasia law itself” (De Morgen, December 1, 2019).*

*“Worse is that Brother (name of the Brother), Superior General of the Brothers of Charity, may not be interrogated as a witness” (De Tijd, December 19 2019).*

*“This is a strange trial, where the counsellors, oh wonderful coincidence, previously represented bishop (name of the bishop) and cardinal (name of the cardinal) in child abuse cases within the Church*…*and also Superior (name of the Superior) of the Brothers of Charity, in a dispute with his own brothers, which, o wonderful coincidence, concerned the application of euthanasia in the psychiatric institutions of the Order.” (De Morgen, January 25, 2020).*

Newspapers with a historical Christian ideological background [particularly *De Standaard* (C,E)] tried to challenge “these accusations”. In fact, De *Standaard* (C,E) was the only newspaper repeatedly indicating that statements such as *“the highest authorities within the Catholic Church have influenced and directed this process”* were speculations or suspicions, and that pro-euthanasia activists *“should not react nervously as our distaste for normative, religious ethics imposed on us for centuries by the Church runs deep”.*

*“The accusation of influencing remains speculative. We must have faith in Lady Justice. The euthanasia debate has not been a point of discussion between Catholics and liberals for some time now. Among both Catholics and liberals one finds both supporters and opponents” (De Standaard, December 15, 2019, Can we stick to the lawsuit?).*

All newspapers, however, almost exclusively reported on the suspected influence of the Catholic Church. The possible role of freemasonry was only mentioned occasionally in Flemish newspapers. In fact, one of the attorneys of the patient’s family questioned the freemason affiliation of one of the members of the expert commission. Moreover, the latter seemed to have a good understanding with the husband of the consulted psychiatrist, who also used to be a freemason.

“*At the end of their presentation, (name of the attorney), attorney of the (surname of the family) family, had another question: “Can (name of the court-appointed psychiatric expert) confirm that he had been Grand Master of the Regular Grand Loge of Belgium? (De Morgen, January 25, 2020).*

The fact that one of the attorneys defending the consulted psychiatrist also was a member of the Board of Directors of the Life End Information Forum (LEIF), a Flemish organization providing trained consultants in euthanasia requests, is even only mentioned once.

It was also said by newspapers with a historical non-Christian ideological background that the Church would like to create a “chilling effect” by convicting the three physicians to deter other physicians from performing euthanasia in the future.

*“Who has set the agenda behind the scenes will never come to light. The anti-euthanasia lobby has in any case achieved its goal, even if the physicians are not punished” (according to Van Steenbrugge, attorney of the attending psychiatrist) (Het Nieuwsblad, January 23, 2020).*

### Public-social consequences

While some newspapers [particularly *Het Laatste Nieuws* (L,Po), *Het Nieuwsblad* (C,Po), and *De Gazet van Antwerpen* (C,Po)] paid a lot of attention to the possible public-social consequences of this court case, others [i.e., *de Morgen* (S,E)] hardly discussed it. *De Standaard* (C,E) and *Het Belang van Limburg* (C,Po) occupied an intermediate position. The possible feared consequences described by the different newspapers were: legal uncertainty among physicians and patients (leading to a “chilling effect” or a mass withdrawal of physicians from participating in end-of-life decisions due to fear of litigation, and a greater fear among patients that they will lose their “rights”), implications for palliative and end-of-life care and euthanasia legislation (contraction of the law), and a higher number of non-reported cases.

*“In any case, the public pillory to which the doctors are nailed will have the desired deterrent effect. What doctor still dares if ten years later he can still be convicted as an ordinary poisoner?”* (Het Nieuwsblad, January 23, 2020).

*“I think people are afraid that it will no longer be possible in the future. That they will lose their ‘rights’ - as they have sometimes come to regard euthanasia themselves. ‘Doctor, you’re not going to abandon me, are you?’ they ask”* (Gazet van Antwerpen, January 25, 2020).

## Discussion

Our analysis showed that the prominence given to Belgium’s first criminal case concerning euthanasia in most important non-free Flemish newspapers was moderate. Mean prominence scores did not statistically significantly differ between newspapers with a different historical ideological background or form (popular vs. elite). There also seemed to be no difference in the various angles from which Flemish newspapers and magazines approached the euthanasia case. Headline topics most frequently focused on legal aspects, aspects relating to the private or family sphere or personal feelings, and controversial aspects. In general, major Flemish newspapers and magazines, despite their different historical ideological background and form, mostly were neutral in their coverage of the euthanasia case. Nevertheless, our qualitative analysis showed some subtle differences in selection, statement or tonality between the reports of newspapers with a different historical ideological background, despite the fact that all newspapers clearly were opposed to the prosecution of the accused physicians and unequivocally empathetic to the patient’s psychological suffering.

Several news factors influence journalistic and editorial judgments of a story’s newsworthiness or prominence, including conflict (the presence of disagreements and/or controversy between people or institutions), eliteness (the presence of people or institutions of elite status involved in an event), proximity (or geographical or psychological ‘nearness’ of an event), and consequence (an event causing a great sequence of events affecting many people) (27, 28). Although one should also account for the psychology of news decisions made by editorials or for the role of ideology, some of the abovementioned factors more than likely were relevant for the prominent news coverage of this case. An important one certainly was the potential consequences a conviction of the physicians would have for euthanasia legislation and the willingness of physicians to perform euthanasia for psychiatric patients in the future.

Several newspapers [particularly *Het Laatste Nieuws* (L,Po), *Het Nieuwsblad* (C,Po), and *De Gazet van Antwerpen* (C,Po)] paid a lot of attention to the possible public-social consequences of this court case. Although the media exposure of the euthanasia case certainly has led to a greater awareness of, and sensibility toward the rights of non-terminally ill patients among the general public, giving a face to the suffering of psychiatric patients desiring to end their lives, it also seemed to have had a major impact on medical care providers. Already during the trial it was clear that the judicial procedure made physicians in Belgium nervous about signing papers for patients or helping them die. On the other hand, patients were anxious that physicians would not be willing anymore to fulfill their request to die. According to a survey of the Belgian Physicians Journal *De Artsenkrant* (*n* = 776), that has been conducted shortly after the court trial, one third of the Belgian physicians now speak out against euthanasia for people who are not terminally ill and want a contraction of the law, 70% want an evaluation of the Belgian Euthanasia Law, and half of them a revision of the FCECE (29). According to a survey conducted by the LEIF in February 2021 (*n* = 181), 55% of the physicians participating in end-of-life decisions are less willing to perform euthanasia for psychiatric disorders nowadays (compared to 31% before the judicial case), and one third of these physicians refuse to give advice (compared to one fourth of the physicians before the judicial case) (30). These reservations among certain professionals, however, have been around for some time. In December 2015 *De Morgen* (S,E) published an open letter by 65 professors, psychiatrists and psychologists, including one of the experts appointed by the civil party during the judicial case, calling for the 2002 Euthanasia Law to exclude euthanasia for psychological suffering. Very recently, several newspaper reports and surveys indeed indicated that things have changed since the case. More physicians nowadays refuse to participate in end-of-life decisions due to fear of litigation (criminal charges for murder) or because they are intimidated by relatives of people who were or are being to be euthanized, or by their attorneys. Families opposing requests for euthanasia and engaging attorneys to dispute the issue, seems to be a new development after the case. It turns out that physicians dread the high risk of prosecution. Physicians signal that they do not want to be dragged into the criminal justice system because of helping this kind of patients.

Although major Flemish newspapers and magazines generally were neutral in their coverage of the euthanasia case, probably indicating that in general newspapers have renounced their original ideological ties, our qualitative analysis nevertheless showed some subtle differences in selection, statement or tonality of reports, particularly between newspapers with a historical Christian-Democratic [particularly *De Standaard* (C,E)] and historical leftist socialist background [i.e., *De Morgen* (S,E)], revealing value judgments when describing the persons involved in the euthanasia case and framing legal and religious-political issues related to the case. While the major newspaper *De Standaard* (C,E) seemed to emphasize the problematic nature of euthanasia due to psychiatric suffering, by discussing critically and extensively difficult issues such as the uncertainty in psychiatric diagnosis (diagnostic ambiguity in psychiatry and the pivotal issue of “irremediability” in the context of mental diseases) or the problematic functioning of the FCECE, it seems that the major historical socialist newspaper *De Morgen* (C,E) was trying to prove the opposite. Different Flemish newspapers also portrayed the accused physicians differently. Although all newspapers were critical for the attending physician, particularly *De Morgen* (S,E) equally defended the physician emphasizing that his bad reputation doesn’t make him a murderer, sometimes even calling him courageous. *De Standaard* generally was also more critical for the other accused physicians, compared to other Flemish newspapers (e.g., one of the articles that appeared in *De Standaard* (C,E) was titled *“When physicians open their umbrella”*, meaning avoiding one’s responsibility, and letting everyone else take the blame). While several newspapers with a historical non-Christian ideological background insinuated that this trial indeed was orchestrated by the “Catholic Lobby” with the intention to roll back the law and to induce a “chilling effect” among physicians, the newspaper *De Standaard* (C,E) indicated that all this is speculation.

Particularly *De Standaard* (C,E) extensively reported on the lack of consensus among experts of the parties on the diagnosis issue. One cannot deny that diagnostic ambiguity exists in psychiatry. Although autism spectrum disorders (ASDs) and BPDs are accepted by the American Psychiatric Association as two distinct disorders, a symptomatic overlap between both disorders may exist (31, 32), often making differential diagnosis difficult and challenging. Asperger’s Syndrome, one of the diagnoses for which the patient was granted euthanasia and which is nowadays known as high functioning ASD (without intellectual disability), appears to share various features of BPD (32) (for more information on this issue we refer the reader to De Hert et al. (5). This partly explains why some females with BPD have undiagnosed ASD. Moreover, a lack of comprehensive assessment and knowledge on autism in mental health services may further contribute to the higher risk of misdiagnosis (33). Examining the differential diagnosis of ASDs and BPDs only became the focus of research in recent years. This research is important as there is some indication that this comorbid population may have an elevated suicide risk (34, 35). Moreover, autism that has not been diagnosed in childhood, as has been suggested to be the case for the patient, may lead to higher levels of stress in these individuals when trying to find a lifestyle to survive in a world that is difficult to understand (31).

### Critical reflection

Legal ambiguities more than likely led to the subtle differences in selection, statement or tonality between newspapers. The National Council of the Order of Physicians in Belgium, as well as most Flemish newspapers and magazines reporting on the trial suggested that the judicial case indicated the need for an evaluation and possible amendments to the existing Euthanasia Law, without touching the people’s right to self-determination. Recently, judgments of the European Court of Human Rights (ECHR) and the Constitutional Court of Belgium reinforced this need for legal reform. In the case brought before the ECHR (*Mortier v. Belgium*), concerning the euthanasia of a 64-year old Belgian women with treatment-resistant depression and a personality disorder, the Court ruled that the *a posteriori* control of the euthanasia case was inadequate. According to the ECHR, the control system established in this case did not ensure its independence and therefore was seen as a violation of human rights (i.e., article 2, guaranteeing the protection of the right to life). In fact, the anonymous part of the registration document allows a physician who sits on the FCECE to remain silent and to vote on the legality of a euthanasia case in which he or she has been involved. This ruling calls on the Belgian legislature to revise the control system in a way that can guarantee the independence of the FCECE in each individual case. When considering this point, the Belgian legislature could take inspiration from the control mechanism in the Netherlands, where reporting is not anonymous. The case brought before the Belgian Constitutional Court concerned the one that has been discussed in this article. The fact that the attending physician is still facing a civil trial led the responsible judge to put preliminary questions to this Court about the sanctions that apply to breaches of the Euthanasia Law. As this Law does not contain specific sanctions, the general provisions of the Criminal Code apply, resulting in a situation that any infraction, even a non-essential, administrative error, would amount to murder. Therefore, the Constitutional Court concluded that the system of penalties for physicians who do not fulfill the legal due care criteria when performing euthanasia for psychiatric patients is a violation of the constitutional principles of equality and non-discrimination (36, 37).

Other recommendations have recently been made by De Hert et al. (5) and the Flemish Psychiatric Association (38). In fact, another important point of contention concerns the absence of any consensus or guidance on how to define psychological suffering, making it possible to use the concept in an increasingly broad way (17). In December 2017, The Flemish Psychiatric Association (FPA) (38), founded in 2004, published its advisory text on ‘How to handle a patient request for euthanasia in psychiatry under current law?’ with the purpose to provide additional legal safeguards. Although the “persistent and unbearable physical or psychological suffering that cannot be alleviated” is primarily subject to an assessment by the patient itself, the FPA in their guideline for euthanasia stressed that unbearableness should also be evaluated by the physician/psychiatrist through “repeated consultations, thorough observation and examination of the patient” (p. 21). According to this guideline all indicated biological and psychotherapeutic treatments, as well as social interventions that may alleviate the suffering must have been tried and have been found to be ineffective before a patient with a psychiatric disorder can be considered “incurable” and qualifies for euthanasia (p. 17). This means that in case of treatment refusal, which is a fundamental moral right and is enshrined in the 2002 Belgian Law on Patients’ Right, the legal requirement of the “incurability” of the disease cannot been shown and therefore, contrary to the FCECE (for whom patients may refuse curative treatments if the side effects of those treatments are deemed unbearable to the patient) (13), disqualifies the patient for euthanasia. The Flemish guideline also recommends for euthanasia performed on psychiatric patients a time-period of at least 1 year (instead of 1 month, as is indicated by the law) between the written request and the actual performance of euthanasia (p. 12), that at least two independent psychiatrists should be involved (p. 15), and that during the evaluation a ‘twin-track policy’ (one evaluating the euthanasia request, the other exploring all remaining evidence-based therapeutic options) should be a fundamental attitude (p. 9). At least two independent and positive advices are necessary (p. 14) and the family should be maximally involved in the process (p. 27).

One of the reasons for these legal shortcomings has to do with the absence of a thorough evaluation process of the Belgian Euthanasia Law. The Euthanasia Law in The Netherlands has already been evaluated three times to see whether all actors are satisfied with the content and functioning of the law. In contrast, although various legal amendments have been made since the Belgian Euthanasia Law came into force in 2002, the due care criteria of the Law actually haven’t been evaluated by an expert commission in the 20-year time period that has passed (despite the fact that it has been suggested and planned).

Before the decriminalization of euthanasia in the Benelux countries (Belgium, Netherlands, and Luxembourg) research already indicated a significant increase in euthanasia acceptance among the general public in most West European countries during the time period 1980–1999. Particularly Belgium drastically changed its acceptance of euthanasia, which probably has been a major contribution to the Belgian euthanasia legislation of 2002 (18), making Belgium the second country in the world to legalize the procedure, after the Netherlands. This increase in euthanasia acceptance was found to be strongly associated with the particularly strong decrease in religious beliefs and a greater emphasis on the individual’s right to self-determination. For many centuries Flanders (and Belgium) has been a region dominated by Catholicism. However, since the 1970s, church attendance in Belgium declined tremendously. The accession of the so-called “purple” coalition government, made up of the socialists, the liberals and the green parties (thus without the participation of the moderate-right Christian Democrat party who after more than 50 years in power had suffered a historic defeat) in the 1999 elections signified a clear break from the catholic colored political tradition (39, 40). In 2002 in Belgium a specific law was introduced to determine under what conditions a physician may carry out euthanasia without falling under the scope of criminal law.

The political agreement to put the individual’s right to self-determination at the end-of-life into a legal framework did not imply that euthanasia as a practice was not contested. This content analysis of Flemish newspapers and magazines shows that the acceptability of medical interventions in the dying process of people who are not terminally ill continues to be highly controversial. The emotional descriptions of the persons involved in Belgium’s first criminal case concerning euthanasia for psychiatric patients indicates that is it difficult to detach the discussion from personal and emotional attitudes. The main arguments on each side of the euthanasia debate that have been uncovered by this content analysis are similar to those found globally. In fact, global arguments against euthanasia include religious and/or moral objections and concerns about physician errors (diagnostic) or about the abuse/coercion of vulnerable people (41, 42). The autonomy/right to die argument, as well as the argument “pushing for compassion and the relief of suffering” features prominently in the reasoning of people who are for euthanasia. A recent systematic review (43) among older adults (≥60 years) identified lower religiosity as one of the most consistent predictors associated with positive attitudes toward MAID. Significant correlations have also been found between attitudes in support for MAID and psychological constructs like conscientiousness and neuroticism (44). It has been suggested that attitudes or opinions of people have changed over time and became more liberal as a result of increased media and legal focus on euthanasia over the past few years. This probably explains that while several studies (44–48) have shown that religiosity predicts more negative attitudes, even among healthcare professionals, other studies [e.g., (49)] have contradicted this. Moreover, major religions vary dramatically in terms of their views on MAID. Perspectives may vary even within the same religion based on the subgroup to which the patient or provider belongs, or even their country of residence (50). This illustrates the complexity of attitudes toward MAID. Nevertheless, societal changes, such as a decline in the influence of the church on society, advances in healthcare and medical technology, a growing population of older adults, and an increase in emphasis on the individual may all contribute to an environment in which more positive public attitudes toward euthanasia could progress (42).

### Strengths and limitations

This content analysis combined qualitative with quantitative research strategies to look at the media coverage of Belgium’s first criminal case concerning euthanasia for psychiatric patients and to evaluate differences in selection, statement or tonality of reports, adding valuable nuances which otherwise would have been missed in solely quantitative data collection. Moreover, until now, few studies have focused on the media coverage of MAID for people who are not terminally ill, such as those suffering from psychiatric disorders, which serves as an important base to identify and analyze the presence of relevant themes about media attention and community response, particularly within a country that has already since the last decennia of the 20th century drastically changed its acceptance of euthanasia.

Nevertheless, we need to mention certain limitations. This content analysis solely focused on *Flemish* print and online newspapers and magazines, as previous research [e.g., (11, 16)] revealed significant differences in the practice, knowledge, and attitudes regarding euthanasia and its legal requirements between different Belgian regions. The results of this analysis therefore cannot be seen as reflecting the *Belgian* newspaper industry or community. Another limitation is that the coding of the articles, as well as the thematic analysis was performed by only one of the authors (JD). Despite this, the coding system was discussed and pretested extensively during several online meetings with a multidisciplinary team including five of the authors. Finally, our research questions drove the analysis. Therefore, different research questions could result in new themes. However, as the most important themes have been identified (which has been confirmed by the results of our quantitative analysis), data saturation was sufficient.

## Conclusion

Most major Flemish newspapers and magazines, despite their different historical ideological background and form, gave equal prominence to Belgium’s first criminal case concerning euthanasia for psychiatric patients, approached the case using the same angles, and mostly were neutral in their coverage of the judicial case. Nevertheless, our qualitative analysis showed some subtle differences in selection, statement or tonality of reports on the euthanasia case and the persons involved in this case between newspapers with a different historical ideological background. These discrepancies show that euthanasia based on psychiatric diagnosis and suffering raises complex clinical, ethical and legal dilemmas, partly due to legal ambiguities and the lack of clarity in psychiatric diagnosis assessment, partly to procedural difficulties (such as the problematic functioning of the FCECE), and indicate the need for an evaluation and possible amendments to the existing Euthanasia Law.

## Data availability statement

The original contributions presented in the study are included in the Supplementary material, further inquiries can be directed to the corresponding author.

## Author contributions

MD: conception. KV: statistics. JD: writing first draft of the manuscript. All authors were involved in the study design, execution, and final draft of the manuscript and approved the submitted version.

## Footnotes

1 After March 17, 2023, MAID on the grounds of unbearable suffering caused by a psychiatric disorder will also be legally possible in Canada.

2 The number of requests by patients with a psychiatric disorder is much higher, but 90% of the requests do not end in euthanasia.

3 The original euthanasia request was made on the 22nd of December 2009.

4
https://www.gopress.be

5 Although these concepts sometimes are characterized by fuzziness and fluid boundaries (22), it is a division that is usually made in the (Belgian) newspaper industry. In elite newspapers, the focus is more on (inter)national, economic and political news, while popular newspapers spend more attention to human interest, criminal news, and entertainment. The free newspapers contain only short factual articles—which are often taken literally from the Belga News Agency.
