# Supplementary material for: Media coverage of Belgium’s first criminal case concerning euthanasia for psychiatric patients: A content analysis of Flemish newspapers and magazines
Source: Front Psychiatry. 2023 Jan 4;13:1050086. doi: 10.3389/fpsyt.2022.1050086 (PMC9845880; doi:10.3389/fpsyt.2022.1050086)

**CODING SHEET**

| **1** | **NAME OF NEWSPAPER, MAGAZINE, OR ONLINE NEWS SERVICES OF THE FLEMISH PUBLIC BROADCAST OR THE BELGA NEWS AGENCY** | **NEWSPAPER/ MAGAZINE**  De Morgen  De Standaard  De Streekkrant  De Tijd  De Zondag  Gazet van Antwerpen  Het Belang van Limburg  Het Laatste Nieuws + De Nieuwe Gazet  Het Nieuwsblad + De Gentenaar  Krant van West-Vlaanderen  Metro NL  Knack magazine  Humo  **FLEMISH PUBLIC BROADCAST OR THE BELGA NEWS AGENCY**  VRT online  Belga |  |
| --- | --- | --- | --- |
| **2** | **PUBLICATION DATE** |  |  |
| **3** | **AUTHOR (GENDER, Male/Female)/TITLE OF ARTICLE/ TYPE OF ARTICLE** | Regular news report, letter(s) to the editor, column, expert opinion, comment or other stories/articles providing background information |  |
| **4** | **TOPIC(S) FEATURED IN THE HEADLINE**  **(See also Figure Q7 “Topic(s) featured in the headline” for definitions of all headline topics)**  **HEADLINE TONE**  **(See also Figure Q8 “Headline tone” for definitions of all categories)**  **What is the tone of the headline in general? (Q8A)**  **What is the headline tone toward the euthanasia case? (Q8B)** | Legal aspects  Ethical aspects  Personal/Familial aspects  Political aspects  Controversial aspects  Medical or scientific aspects  Public and social aspects  Religious aspects  Historical aspects  Other  Positive  Negative  Neutral  Positive  Negative  Neutral  Not applicable | If the headline features several aspects of the case it is coded as many times as appropriate to the data (rich coding strategy)  “Proces over euthanasie kan wet openbreken” (De Tijd, 15 januari 2020)  “Laat volksjury euthanasiewet zachtjes aan inslapen?” (De Morgen, 14 januari 2020)  “Euthanasieproces: Geens wil wet aanpassen” (De Morgen, 27 januari)  “Vrijspraak? Dan doen dokters helemáál wat ze willen” (HLN, 30 januari 2020)  “Een publiek proces is in deze emotionele zaak compleet ongepast voyeurisme” (HLN, 21 januari 2020)  “Respecteer de wens om waardig te mogen sterven” (Gazet van Antwerpen, 21 januari 2020)  “Haar armen maakten nog een beweging. Mama riep “Ze wil het niet! Ze wil het niet!” (HLN, 22 januari 2020)  “Tine Nys. Of hoe een prachtmens wordt verfrommeld tot een ‘zaak’” (De Morgen, 20 januari 2020)  “Drugs, prostitutie, veel slaag. En een hardnekkige doodswens” (Het Nieuwsblad, 18 januari 2020)  “’Voor Tine was leven lijden met lange ij’” (De Standaard, 18 januari 2020)  “Pijnlijk proces zet politici aan het werk” (De Standaard, 1 februari 2020)  “Dokters zijn er toch om te genezen, niet om te helpen sterven?” (Gazet van Antwerpen, 28 januari 2020)  “Dat mens kon niet meer” (HLN, 21 januari 2020)  “Het leek soms wel het autismeproces” (Het Nieuwsblad, 1 februari 2020)  “Autisme? Borderline? Allebei?” (HLN, 24 januari 2020)  “Palliatieve zorg, de grote verliezer” (De Standaard, 21 januari 2020)  “Stadhuis wordt weer even rechtbank” (HLN, 13 januari 2021)  “Tele-Onthaal krijgt dubbel zoveel oproepen” (HLN, 1 februari 2020)  “Huisartsen trekken zich terug uit euthanasiedossiers” (De Standaard, 31 januari 2020)  “Gelovigen mogen ook strijden voor hun overtuigingen” (De Standaard, 22 september 2020)  “Niemand verwacht de Spaanse inquisitie” (De Standaard, 25 januari 2020)  ‘Het waren de beginjaren van euthanasie bij psychisch lijden' (De Standaard, 20 januari 2020)  Standpunt (De Morgen, 13 februari 2020) |
| **5** | **PROMINENCE SCORE (prominence can be operationalized by a story’s relative position and by the size of the story)**  **(See also Table 2 “Prominence score for coding databases” of the article)** | Numerical rating, ranging from 3 to 16 points, based on the following criteria:  **Placement** **in the newspaper** [front page of first section, front page of inside section, inside prominent (e.g., editorial), or other];  **Headline word count;**  **Length of article word count;**  **Presence or absence of photos (with/without caption).**  Articles that have a higher number of assigned points are considered to be more ‘‘prominent,’’ more likely to attract the ‘‘attention’’ of readers |  |
| **6** | **ARTICLE DIRECTION TOWARD THE EUTHANASIA CASE**  **(See also Figure Q9 “Article direction toward the euthanasia case” for definitions of all categories)** | Favorable  Balanced/neutral  Unfavorable  Not applicable | This score is derived from an evaluation of the article content, using the entire article as a sampling unit. A major challenge is clarifying the article’s perspective on the euthanasia case, keeping this issue separate from what might sometimes be the same article’s perspective on a key ‘‘media figurehead’’.  Coverage deemed favorable to euthanasia includes those articles that framed this act as an essential moral human right, even describing it as the ‘‘ultimate humanistic choice.’’ In many cases, these articles will argue that euthanasia is a right and that nobody has the right to get involved with a decision this personal. An article could also be considered favorable if it was sympathetic to the many organizations that support voluntary euthanasia or support doctors who aid in the process.  Coverage unfavorable to euthanasia included articles using such phrases as ‘‘assisting suicide is the first step towards barbarism,’’ or ‘‘no doctor has the right to play God.’’ Unfavorable articles were quite directly opposed to physician-assisted suicide, stating such reasons as basic morals, ethics, and religious stances. Articles stating that any doctor who would consider aiding a person seeking to end his life was violating the Hippocratic oath, or stressing improved end-of-life care instead of assisted suicide, were also considered unfavorable.  Balanced/neutral coverage included articles that displayed both sides of the debate over physician-assisted suicide in approximately equal measure. Those articles that took the ‘higher moral ground approach,’’ stating that this issue is not for any of us to judge, were also considered neutral. Finally, articles that dealt solely with Dr. Jack Kevorkian and did not render a clear opinion on the more general topic of physician-assisted euthanasia were deemed balanced/neutral. |
| **7** | **MEDIA PORTRAYAL OF MAJOR PERSONS INVOLVED IN THE EUTHANASIA CASE** | **The patient**   1. Newspaper articles giving a dramatic pseudoscientific and emotional description of the patient’s condition, making her a **tragic figure** **described in terms of her illness and in terms of her extreme suffering** 2. Newspaper articles making her **a victim of psychiatry and advocates of euthanasia**, stating that she did not want to die, that this is not what she really wanted, that there was a pressure to ask for euthanasia, that she might have changed her mind. 3. Newspaper articles describing the patient as electing death as a good and calm ending to her suffering, **making her heroic,** **a courageous warrior, leaving a legacy of choice.** 4. Newspaper articles describing the patient as **a** **normal person enjoying live, as an angel, helping other people, as a driven person with dreams like everybody else.**   **The consulted psychiatrist**   1. Newspaper articles describing her as **someone manipulating and luring suicidal people into their own death** 2. Newspaper articles describing her as someone who did **not take her responsibility** 3. Newspaper articles describing her **as a pionier, as someone who saves people, a courageous warrior** making her heroic   **The attending physician**   1. Newspaper articles describing him as **a murderer, as someone with a bad reputation** 2. Newspaper articles describing him as someone who did **not take his responsibility** 3. Newspaper articles describing him as **someone who did take his responsibility and acted decisively** 4. Newspaper articles describing him **as a clumsy** doctor.   **The (consulted) general practitioner**   1. Newspaper articles describing him as a **primary doctor taking care for patients in the community** 2. Newspaper articles describing him as **a simple man who has been set up**   **The patient’s family**   1. Newspaper articles describing them as **inquisitors** 2. Newspaper articles describing them as **persons who did not take enough care of or did not pay enough attention to the patient** 3. Newspaper articles describing them **as victims of psychiatry and advocates of euthanasia** |  |
| **8** | **LEGAL ASPECTS: DISCUSSION FOCUSING ON GAPS BETWEEN LAW AND PRACTICE** | Incurable disease and unbearable suffering  Diagnostic uncertainty (borderline, autism or both, depressive disorder)  From affirmation that the patient was hopelessly ill and suffering unbearably, that her condition was irremediable and intolerable to the assertion that she did not suffer from a "serious and incurable disorder" and did not want to die, that this is not what she really wanted, that there was a pressure to ask for euthanasia, that she might have changed her mind  Functioning of and reporting to the Federal Control and Evaluation Commission for Euthanasia (FCECE)  Independence |  |
| **9** | **RELIGIOUS-POLITICAL POLEMICS** | Emphasizing the conflict between conservatives (catholics) and liberals (freemasonry) |  |
| **10** | **PUBLIC-SOCIAL CONSEQUENCES** | The possible feared consequences described by the different newspapers: legal uncertainty among physicians and patients (leading to a “chilling effect” or a mass withdrawal of physicians from participating in end-of-life decisions due to fear of litigation, and a greater fear among patients that they will lose their "rights"), implications for palliative and end-of-life care and euthanasia legislation (contraction of the law), and a higher number of non-reported cases. |  |


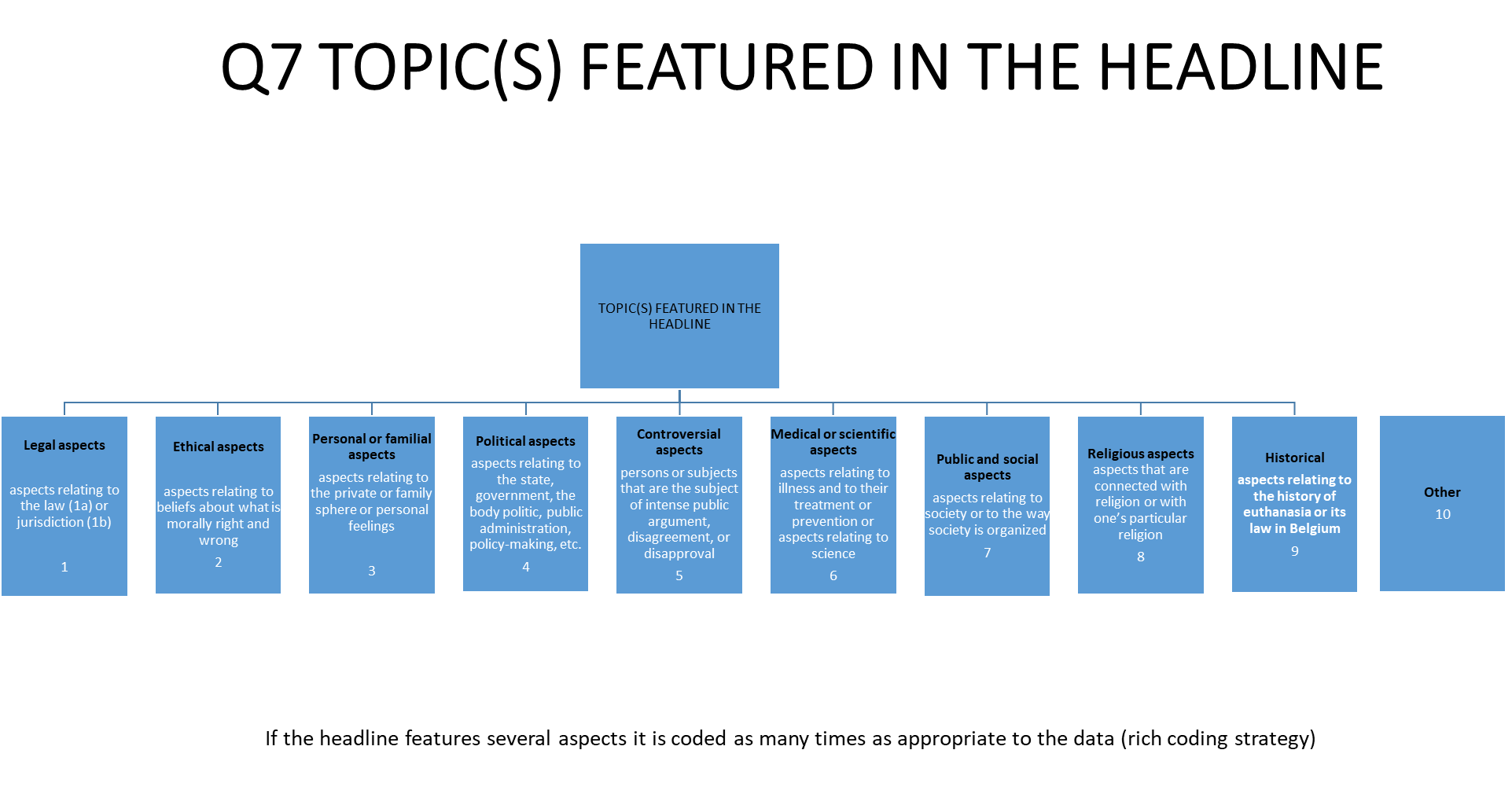


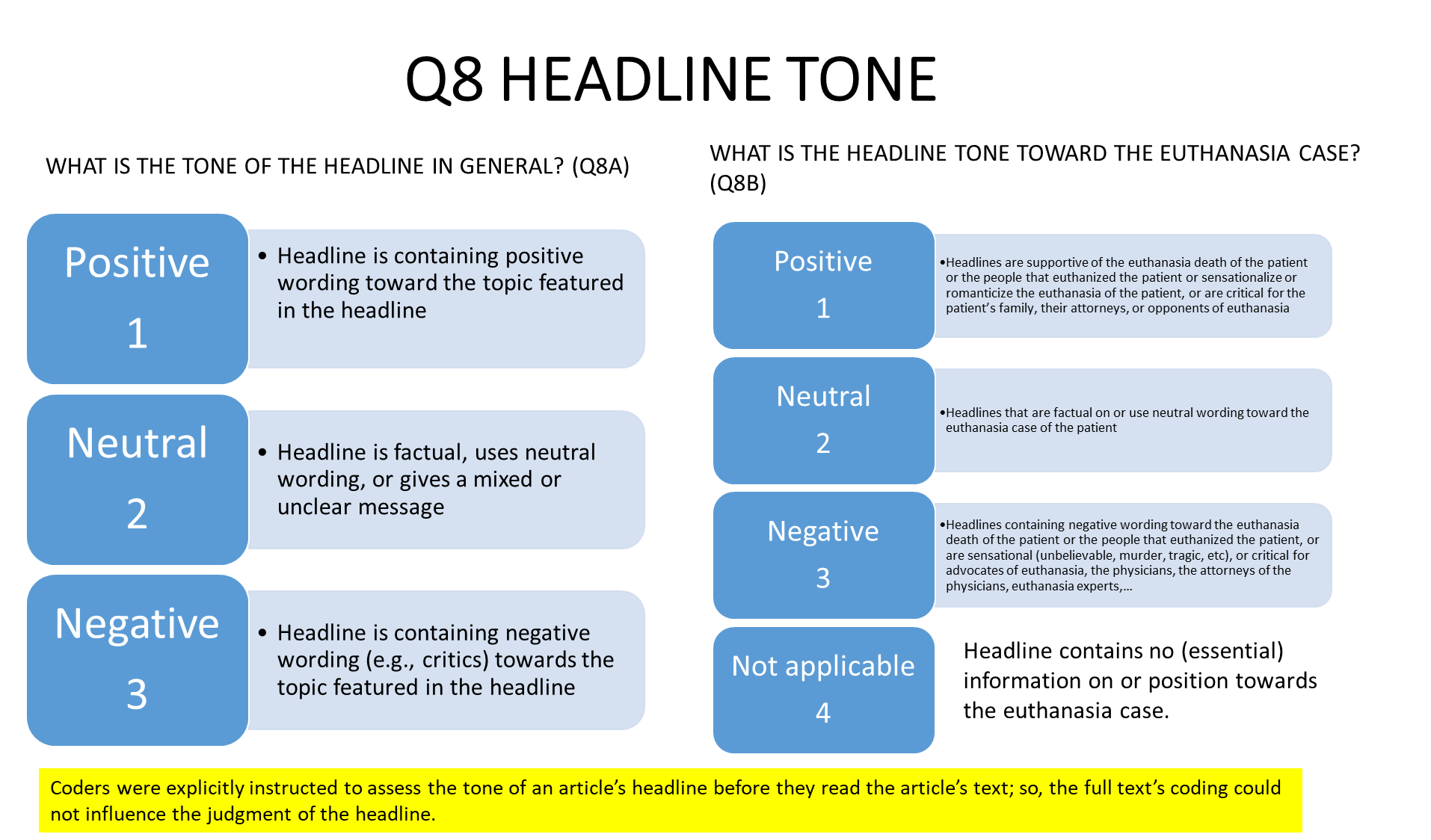


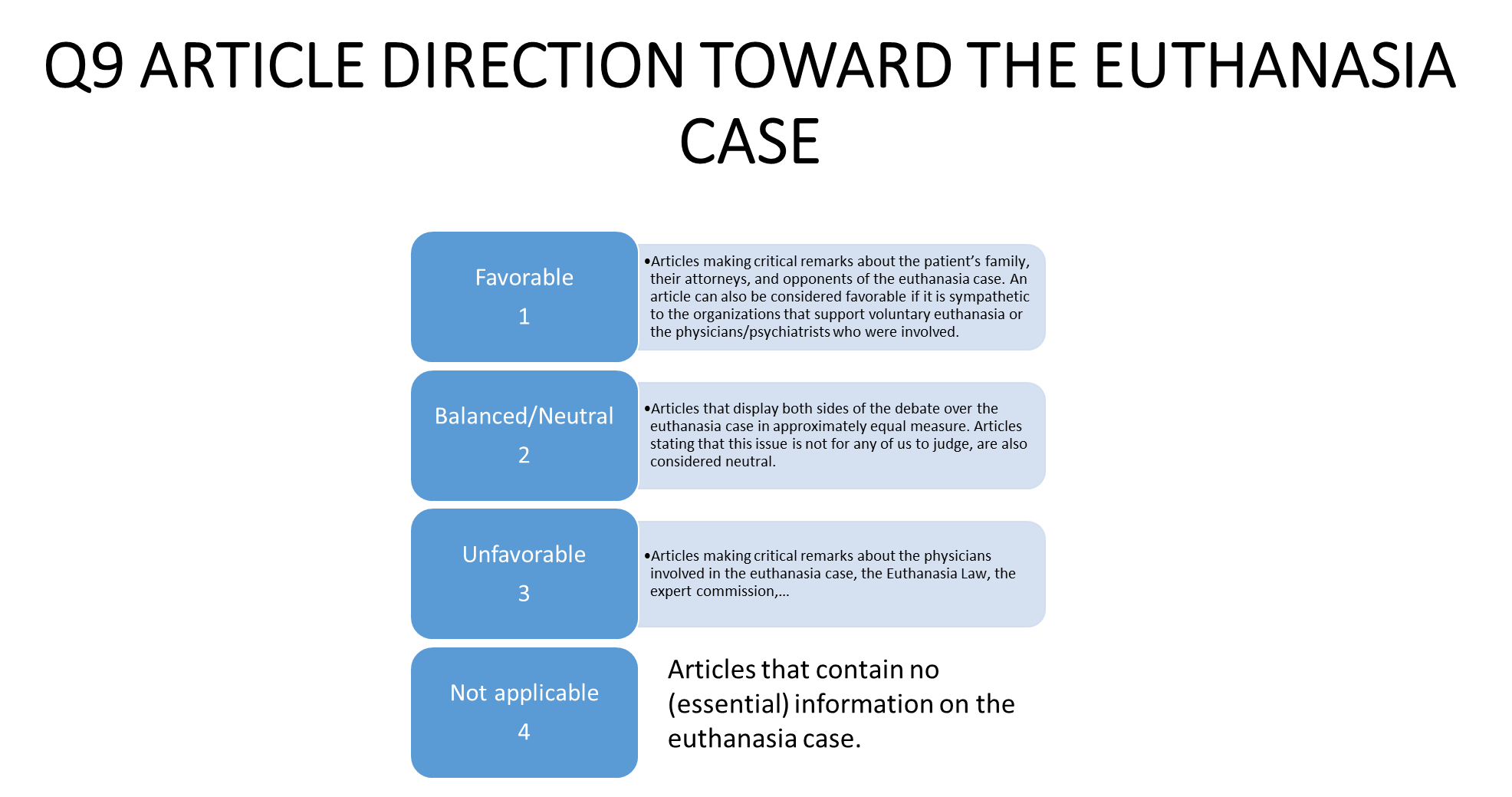

Supplement: Supplementary file 1 [file Data_Sheet_1.docx]
